# Amniotic Fluid Stem Cells from EGFP Transgenic Mice Attenuate Hyperoxia-Induced Acute Lung Injury

**DOI:** 10.1371/journal.pone.0075383

**Published:** 2013-09-11

**Authors:** Shih-Tao Wen, Wei Chen, Hsiao-Ling Chen, Cheng-Wei Lai, Chih-Ching Yen, Kun-Hsiung Lee, Shinn-Chih Wu, Chuan-Mu Chen

**Affiliations:** 1 Department of Life Sciences, Agricultural Biotechnology Center, National Chung Hsing University, Taichung, Taiwan; 2 Division of Pulmonary and Critical Care Medicine, Chia-Yi Christian Hospital, Chia-Yi, Taiwan; 3 Department of Bioresources and Molecular Biotechnology, Da-Yeh University, Changhwa, Taiwan; 4 Division of Pulmonary and Critical Care Medicine, China Medical University Hospital, Taichung, Taiwan; 5 Division of Biotechnology, Animal Technology Institute Taiwan, Miaoli, Taiwan; 6 Department of Animal Science and Technology, National Taiwan University, Taipei, Taiwan; University Heart Center Freiburg, Germany

## Abstract

High concentrations of oxygen aggravate the severity of lung injury in patients requiring mechanical ventilation. Although mesenchymal stem cells have been shown to effectively attenuate various injured tissues, there is limited information regarding a role for amniotic fluid stem cells (AFSCs) in treating acute lung injury. We hypothesized that intravenous delivery of AFSCs would attenuate lung injury in an experimental model of hyperoxia-induced lung injury. AFSCs were isolated from EGFP transgenic mice. The *in vitro* differentiation, surface markers, and migration of the AFSCs were assessed by specific staining, flow cytometry, and a co-culture system, respectively. The *in vivo* therapeutic potential of AFSCs was evaluated in a model of acute hyperoxia-induced lung injury in mice. The administration of AFSCs significantly reduced the hyperoxia-induced pulmonary inflammation, as reflected by significant reductions in lung wet/dry ratio, neutrophil counts, and the level of apoptosis, as well as reducing the levels of inflammatory cytokine (IL-1β, IL-6, and TNF-α) and early-stage fibrosis in lung tissues. Moreover, EGFP-expressing AFSCs were detected and engrafted into a peripheral lung epithelial cell lineage by fluorescence microscopy and DAPI stain. Intravenous administration of AFSCs may offer a new therapeutic strategy for acute lung injury (ALI), for which efficient treatments are currently unavailable.

## Introduction

Acute respiratory distress syndrome (ARDS) is a condition characterized by acute onset, bilateral lung infiltrates, refractory hypoxemia, and the absence of cardiogenic pulmonary edema. ARDS and acute lung injury (ALI) are major causes of mortality and morbidity in critically ill patients [1,2]. A cohort study in 21 hospitals in the United States revealed the age-adjusted incidence of ALI to be 86.2 per 100,000 person-years with 40% in-hospital mortality [3]. Clinically, ALI may be the end result of several disorders, such as pneumonia and pulmonary contusion, that directly injure the lung or those that indirectly injure the lung, including sepsis, severe trauma, and transfusions of blood products [4,5]. Histologically, the acute exudative phase (the first 24-72 h) of ALI is characterized by infiltration of inflammatory cells and disruption of the alveolar–capillary barrier, leading to a proteinaceous exudate that floods the alveolar spaces and then impairs gas exchange and precipitates respiratory failure [6,7].

Patients with ALI may require mechanical ventilation support with a high concentration of inspired oxygen. However, supplemental oxygen can exacerbate the pathogenic processes within the lung [8,9], and this may result in hyperoxia-induced ALI [10,11], the pathological changes of which resemble ARDS in animal models [12]. Thus, therapeutic strategies for hyperoxia-induced ALI may be the same as for clinical ARDS. Despite decades of efforts to identify pharmacologic agents to treat ALI, there are still no effective therapeutic agents for this condition [13].

Bone marrow-derived mesenchymal stem cells (BM-MSCs) can migrate to, or participate in the development of, lung tissue [14,15], and have been shown to have anti-inflammatory effects [16]. Several recent studies have demonstrated that stem/progenitor cells, including mesenchymal stem cells (MSCs), embryonic stem cells (ESCs), genetically engineered stem cells, and umbilical cord MSCs, have the potential to be used as cellular therapies that contribute to lung repair mechanisms after ALI [17–22]. Human amniotic fluid stem cells (AFSCs) have shown the ability to differentiate into lineages belonging to all three germ layers, and appear to have many of the key therapeutic benefits of ESCs while avoiding their ethical drawbacks [23]. Human AFSCs have intermediate characteristics between embryonic and adult stem cells and are able to differentiate into lineages representative of all three germ layers but unlike embryonic stem cells they do not have tumorigenic effect *in vivo* [24]. Furthermore, AFSCs represent an accessible source of cells that can be reprogrammed into pluripotent stem cells with two Yamanaka factors. These characteristics, together with absence of ethical issues concerning their employment, have made stem cells from amniotic fluid a promising candidate for cell therapy and tissue engineering [25].

However, information regarding the use of AFSCs for treating acute lung injury is limited [26]. In this study, we investigated whether murine AFSCs from enhanced green fluorescent protein (EGFP) transgenic mice have beneficial effects on lung function and animal survival in a model of hyperoxia-induced ALI.

## Materials and Methods

### Isolation and culture of murine amniotic fluid stem cells (AFSCs)

The animal use protocol was reviewed and approved by the Institutional Animal Care and Use Committee of the National Chung Hsing University (IACUC Approval No. 101-21). The samples of amniotic fluid (AF) were obtained from pregnant 6-8 week-old, female, EGFP-expressing transgenic mice [27]. Maternal uterine tissue was removed and placed in α-minimal essential medium (α-MEM) supplemented with 10% fetal bovine serum (FBS), then collected AF samples using a sterile insulin syringe with a 29-G x 1/2” needle. The AF samples were centrifuged for 5 min at 1,500 rpm. Cells thus obtained were plated in α-MEM supplemented with 10% FBS and incubated for 48 h at 37 ^o^C containing 95% air and 5% CO_2_. The medium was changed every 72 h, and the cell colonies increased gradually over the next 7 to 10 days [28].

### Colony-forming unit-fibroblast (CFU-F) assay and pluripotency markers detection

The CFU-F potential of suspension-derived cells was determined by plating aliquots (1,000 cells/cm^2^) of cells in complete MesenCult^TM^ medium for 14 days and analyzing the colonies as previously described [29]. Two pluripotency markers, Oct-4 and Nanog, were detected by RT-PCR in original isolates (F-0) and fifth passages (F-5) of mouse AFSCs. The primer sets were designed (forward and reverse, respectively) as follows: *Oct-4*, 5’-GTACTTGTTTAGGGTTAGAG-3’ and 5’-GTGGAAAGACGGCT CACCT-3’; *Nanog*, 5’-AGTGCTCCTTCCAAACCCCA-3’ and 5’-CTGTGCTCC ACGCTGATG-3’. A pair of β-action primer (see below) was used as an internal control.

### Flow cytometry analysis

Cultured cells (passages 5-8) were detached with trypsin/EDTA and counted. Approximately 1 × 10^6^ cells were divided into aliquots in 5-mL centrifuge tubes. The cells were then stained with fluorescent phenyleneethylene (PE)-conjugated rat anti-mouse antibodies: CD44-PE (20 µg/mL, IM7, BD Biosciences, Oxford, UK), or CD105-PE (10 µg/mL, MJ7/18, Abcam, San Francisco, CA, USA), at 25 ^o^C for 30 min. The cells were pelleted, washed twice with PBS and fixed with 1% paraformaldehyde. After fixation, analysis was performed by using a Cytomics FC500 (Beckman Coulter Inc., Fullerton, CA, USA) as described [30].

### In vitro differentiation

Purified cell populations were cultured to 100% confluency and then differentiated by the appropriate inducing media for 21 days. For adipogenesis, the cultured cells were incubated in medium containing α-MEM, 10% FBS, 1 µM dexamethasone, 0.5 mM isobutylmethylxanthine, 10 µg/mL insulin, and 100 µM indomethacin. The cultured cells were then fixed and stained with 0.5% Oil Red O (Sigma-Aldrich, St. Louis, MO, USA). For osteogenesis, the cultured cells were incubated in inducing medium containing α-MEM, 10% FBS, 1 µM dexamethasone, 10 mM glycerol 2-phosphate, and 50 μM ascorbic acid 2-phosphate. The cultured cells were fixed and stained with 2% Alizarin red S (A5533, Sigma-Aldrich) for microscopic examination.

### Murine model of hyperoxia-induced acute lung injury

Eight-week-old female CD-1 (ICR) wild type mice were obtained from the National Laboratory Animal Center (Taipei, Taiwan). The mice were provided with food and water *ad libitum* throughout this study and maintained at a temperature of 25 ^o^C and humidity of 50-70% [30]. The mice were randomly assigned to one of the following four groups (n=10): (1) hyperoxia-exposed mice were treated with AFSCs; (2) hyperoxia-exposed mice were treated with PBS; (3) normoxia-exposed mice were treated with AFSCs; (4) normoxia-exposed mice were treated with PBS. The hyperoxia-exposed mice were housed in humidified 99% oxygen in a hyperoxia chamber with normobaric pressure for 60 h. After 60-h exposure of 99% oxygen, 5 × 10^5^ AFSCs suspended in 100 µL PBS were injected via the tail vein using a sterile insulin syringe with a 29-G x 1/2” needle. The mice were sacrificed at days 1, 3, and 7 after the end of the exposure to the oxygen. For survival rate evaluation, additional two experimental groups received the same dose (5 x 10^5^) of mouse embryonic fibroblast cells (MEFCs) or mouse bone marrow-mesenchymal stem cells (BM-MSCs) were added as the cell therapy controls. All the animal trials were repeated triplicate.

### Histology and immunohistochemistry (IHC)

Lung tissues were fixed in 4% paraformaldehyde overnight, embedded in paraffin, and cut into sections for hematoxylin and eosin (H&E) staining. For IHC analysis, the sections were incubated with primary antibody of goat anti-eGFP (1:200; GeneTex Inc., San Antonio, TX, USA) at 4^o^C for 16 h. After wash with PBS, the sections were double stain with Alexa Fluor^®^ 546 conjugated donkey anti-goat IgG for 1 h. After wash with PBS, the slides were mounted with DAPI-Fluoromount-G^TM^ (SouthernBiotech, Birmingham, AL, USA) and analyzed by confocal microscopy (LSM5l0, Carl Zeiss, Germany) [31].

### Lung wet-to-dry weight ratio analysis

The lung tissues were placed into previously weighed microcentrifuge tubes, and the initial (wet) weight of these lung tissues were measured. After these lung tissues were dried in an oven at 70 ^o^C overnight, the (dry) weight was measured again. The wet/dry ratio was defined as the wet lung mass divided by the dry lung mass.

### Analysis of airway inflammation in bronchoalveolar lavage fluid (BALF)

Bronchoalveolar lavage fluid (BALF) was collected using 500 µL of sterile endotoxin-free saline to wash the lungs. BALF cells were collected by centrifugation at 500 x g at 4°C. The number of BALF cells was determined using a hemocytometer [30].

### TUNEL assay

Apoptotic cells in lung tissue sections were identified as terminal deoxynucleotidyl transferase-mediated dUTP nick-end labeling (TUNEL)-positive cells using a commercial kit (Chemicon, Temecula, CA, USA), according to the manufacturer’s instructions [32].

### Masson trichrome stain for lung fibrosis analysis

The quantity of the collagen fibers in lung tissue sections was evaluated by Masson stain solution as described previously [33].

### RNA isolation and quantitative real-time RT-PCR

Total RNA from the lung tissues from normoxic and hyperoxic mice was isolated using TRIzol reagent (Invitrogen, Carlsbad, CA, USA) and treated with RNase-free DNase I (MBI Fermentas Inc., Lithuania, Germany) to remove any genomic DNA contamination [34]. One microgram of total RNA was reverse-transcribed with MuLV reverse transcriptase and the cDNA was analyzed by real-time PCR using intron-spanning primers. For the quantification of mouse IL-1β, IL-6, and TNF-α mRNA levels, PCR was performed using Brilliant SYBR Green Q-PCR Master Mix (Stratagene, La Jolla, CA, USA) [35]. The results were normalized to β-actin mRNA levels. The primer sequences used (forward and reverse, respectively) were as follows: IL-1β: 5’-GCCCATCCTCTGTGACTCAT-3’ and 5’-AGGCCACAGGTATTTTGTCG-3’; IL-6: 5’-GTTGCCTTCTTGGGACTGAT-3’ and 5’-TGTACTCCAGGTAGCTATGG-3’; TNF-α: 5’-GCCCCCAGTCTGTATCCTTC-3’ and 5’-AGGCAACCTGACCACTC TCC-3; MCP-3: 5'-TCTGCCACGCTTCTGTGCCT-3' and 5'-GCTCTTGAGATTCC TCTTGGGGAT-3'; and β-actin: 5’-ACACCCGCCACCAGTTCGC-3’ and 5’-ACCCAT TCCCACCATCACAC-3’.

### Co-culture experiments

The co-culture method was used to evaluate the impact of AFSCs on the hyperoxia-injured or normal lung tissues *in vitro*. The entire lung was placed in 1 mL α-MEM and then mechanically dispersed to create a suspension. The supernatants from the tissues from hyperoxia-exposed mice or normoxia-exposed mice were placed in the wells of 24-well plate and a 6-µm-pore size membrane trans-well containing 5 × 10^3^ EGFP-expressing AFSCs was inserted into the supernatant-containing wells.

### Statistical analysis

The data are presented as the means ± SD. A Mann–Whitney U-test and Student’s t-test were applied for data analyses. Differences with p<0.05 were considered to be statistically significant.

## Results

### Isolation of AFSCs from EGFP-transgenic mice

**Figure 1:**
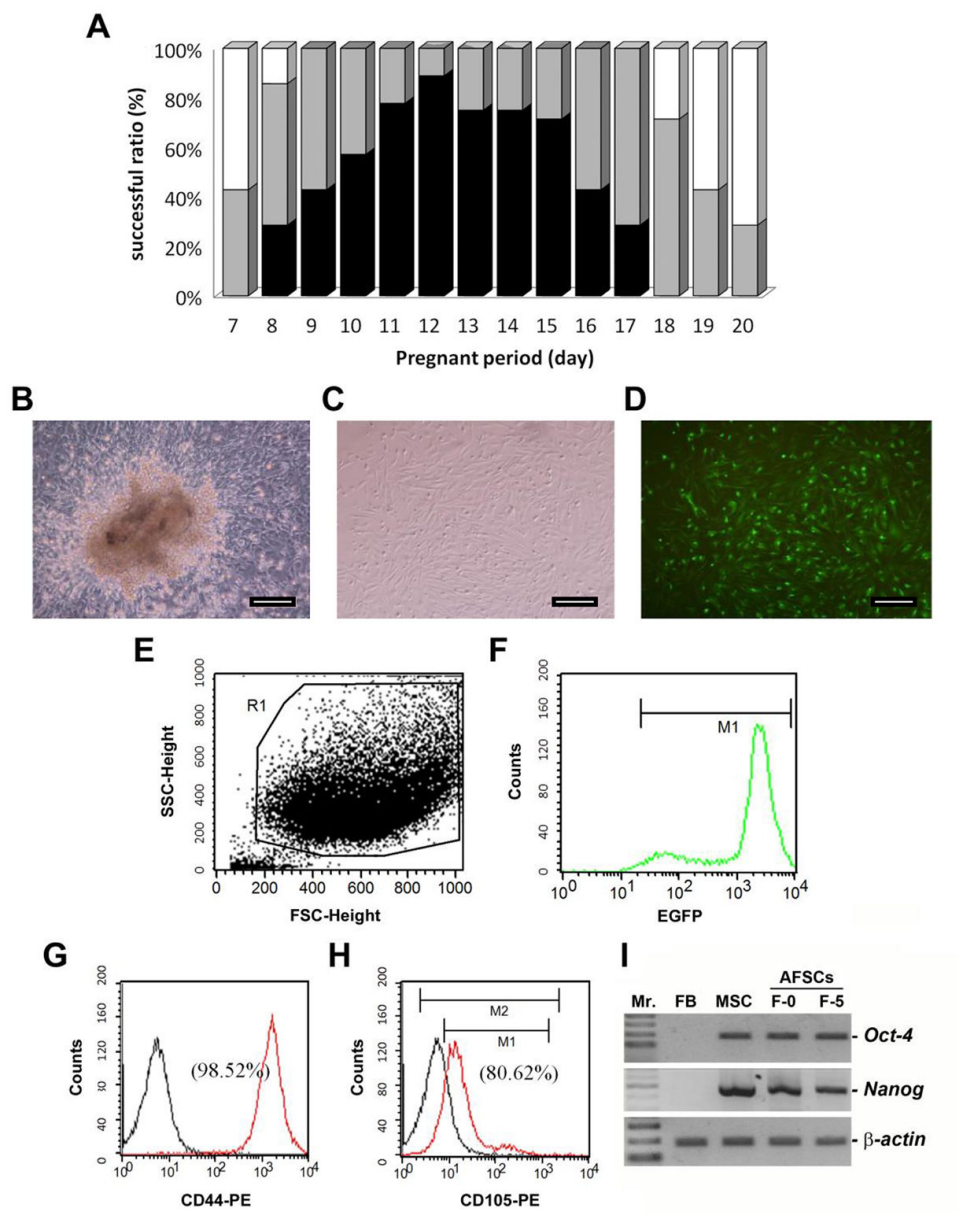
Isolation and characterization of AFSCs from EGFP-expressing transgenic mice. (**A**) The influence of the different pregnancy periods on the success rate of obtaining the murine stem cells from amniotic fluid. The black columns represent the AFSC cells, easily identified after amniotic fluid cell culture and confirmed by the colony forming assay. Grey columns represented that fibroblast-like adherent cells were identified after amniotic fluid cell culture but failed to pass the colony forming test. White columns represented a few cells obtained that were not confirmed to be AFSC cells. n=7 in each examined pregnancy period. (**B**, **C**) The morphologies of identified mouse AFSCs in single layer under bright and fluorescence fields, respectively. (**D**) EGFP-AFSCs expand from the colony after 10 days in culture (100x magnification). Scale bar = 50 µm. (**E**, **F**) Flow cytometric identification of the EGFP-expressing AFSCs. Regions R1 and M1 represent the corresponding fluorescence profile of the EGFP-expressing AFSCs, respectively. (**G**, **H**) Immunophenotypes of EGFP-AFSCs by flow cytometric analysis of the cell surface antigens CD44 and CD105, respectively. Black line represents the control group (unstained cell). Red line represents the cells stained with fluorescent phenyleneethylene (PE)-conjugated rat anti-mouse antibodies with CD44 or CD105. (**I**) Two pluripotency markers of Oct-4 and Nanog in the original isolates (F-0) and fifth passages (F-5) of mouse AFSCs were detected by RT-PCR. Mr.: 100-bp ladder DNA marker. FB: mouse fibroblast cells; MSC: mesenchymal stem cells.

After isolation of the cells from amniotic fluid from mice at gestation stages earlier than Day 7, a few adherent cells were observed, but these cells failed to proliferate. Successful isolation of amniotic fluid stem cells (AFSCs) was achieved from pregnant females between Day 7 and Day 20 of gestation (Figure 1A). Furthermore, the CFU-F test indicated 70-90% higher success rates for the culture of fibroblast-like adherent AFSCs during the middle gestation stages of Days 11 and 12 (Figure 1A).

### Characterization of AFSCs

**Figure 2:**
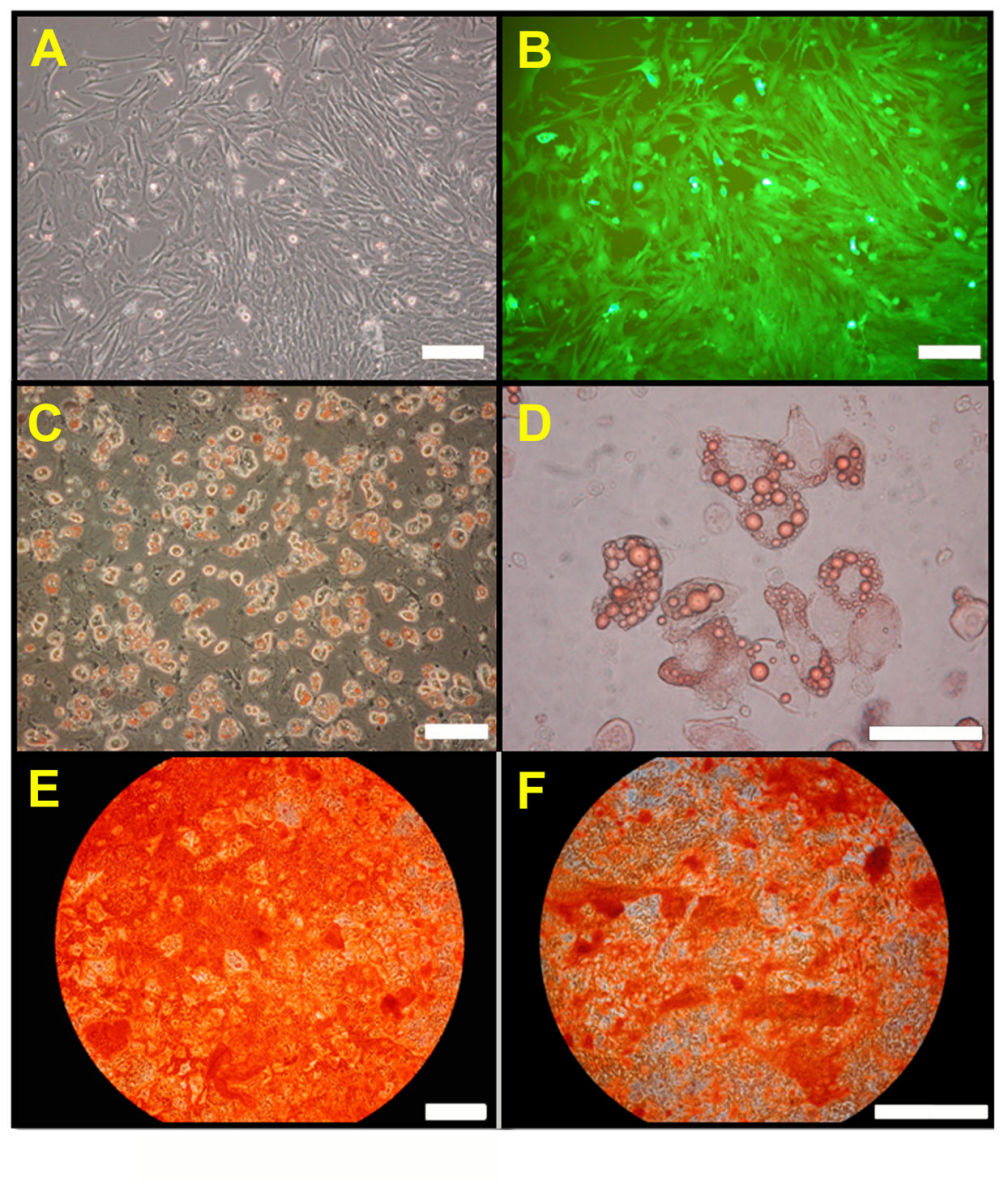
*In vitro* differentiation of EGFP-AFSCs by specific inducing conditions examined by staining. (**A**, **B**) Myofibroblast-like AFSCs expressing EGFP after 7 days in culture under bright and fluorescence fields, respectively. (**C**, **D**) Adipogenic capability of EGFP-AFSCs by Oil Red O staining after three weeks induction, shown at 100x and 200x magnification, respectively. (**E**, **F**) Osteogenic capability of EGFP-AFSCs demonstrated by Alizarin Red S staining after three weeks induction shown at 100x and 200x magnification, respectively. Scale bar = 50 µm.

Colony forming units of AFSCs (Figure 1B) were developed from single EGFP-expressing cells (Figure 1C, 1D). Selected AFSCs by flow cytometry (region R1, Figure 1E) was counted and confirmed for EGFP fluorescent expression (Figure 1F). In Figure 1G and 1H, black line represented the control group (unstained cell). Red line represents the cells stained with fluorescent phenyleneethylene (PE)-conjugated rat anti-mouse antibodies with CD44 or CD105. Flow cytometric analysis revealed that the EGFP-positive AFSCs strongly expressed the cell interaction, adhesion, and migration surface marker CD44 (98.52%; Figure 1G) and moderately expressed CD105 (80.62%; Figure 1H). Two pluripotency markers of *Oct-4* and *Nanog* were also expressed in the original isolates (F-0) and fifth passages (F-5) of mouse AFSCs by RT-PCR analysis (Figure 1I). These results indicated that the cells established from the amniotic fluid were stem cells. *In vitro* differentiation assays demonstrated that the AFSCs from EGFP transgenic mice retained their ability to form osteoblasts (Figure 2C and 2D), and adipocytes (Figure 2E and 2F) at passages 10-15 under the appropriate media conditions. Morphologically, the cells expressed EGFP (Figure 2B) and had a spindled, fibroblast-like appearance in culture (Figure 2A), consistent with the characteristics of AFSC.

### Effect of AFSCs on survival and histopathology of hyperoxia-induced lung injury

**Figure 3:**
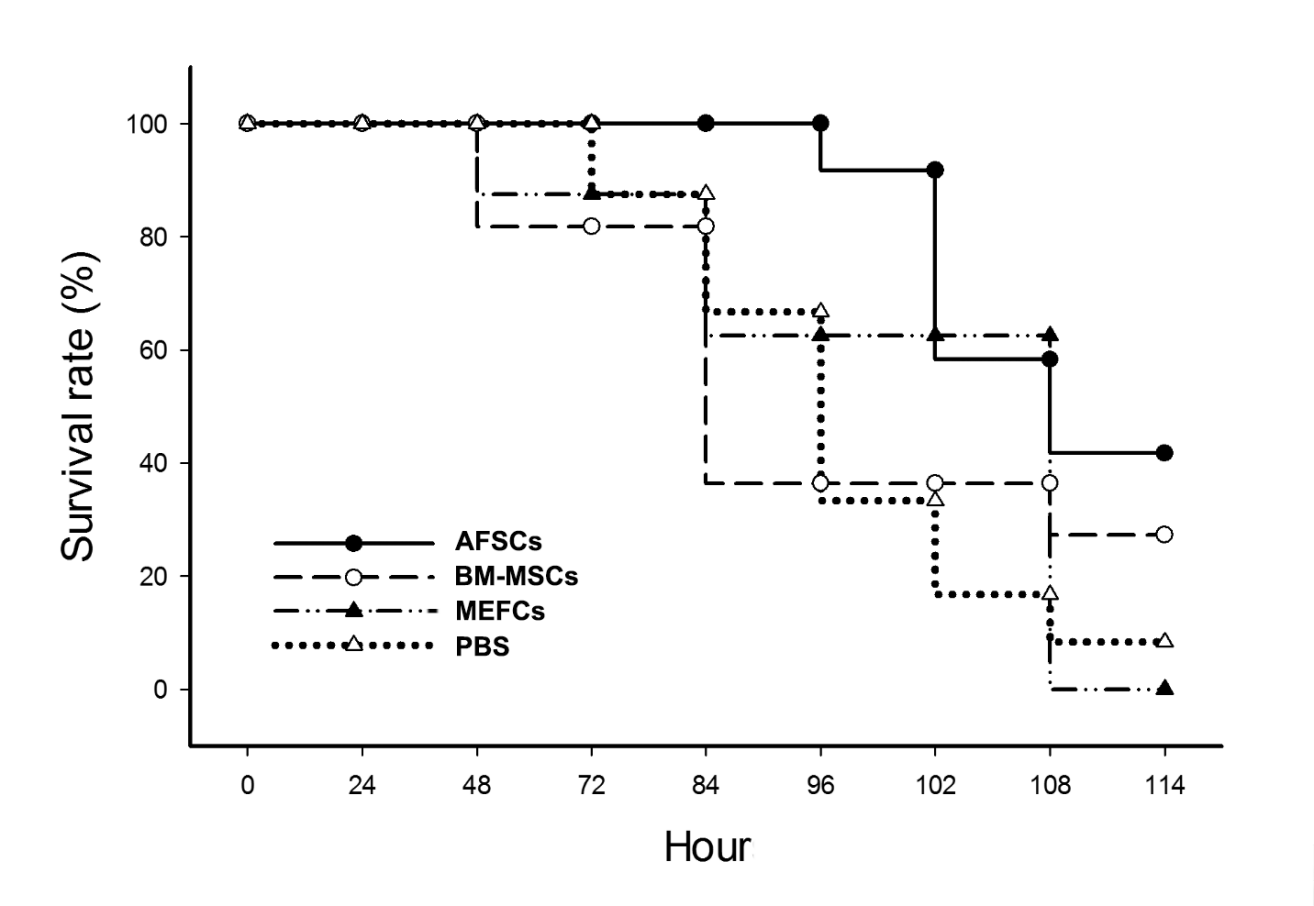
Comparison of the survival rates from the AFSCs-, BM-MSCs-, MEFCs-, and PBS-treated groups. After hyperoxia exposure for 60 h, mice were randomly separated in four groups which received intravenously 5 x 10^5^ AFSCs or BM-MSCs as the test groups (n=10), MEFCs as a control group (n=10), and PBS treatment as a placebo control group (n=10). The survival rates were observed and plotted at 6 h or 12 h intervals. The experiments were repeated three times.

None of the mice died during the first 60 h of exposure to hyperoxia (hyperoxia-exposure period). These mice were randomly separated in four groups (n=10), one of which received the therapeutic intervention of 5 x 10^5^ AFSCs (test group), second and third groups received the same dose of mouse bone marrow-mesenchymal stem cells (5 x 10^5^ BM-MSCs) and mouse embryonic fibroblast cells (5 x 10^5^ MEFCs), respectively, and fourth group received the PBS placebo as a control. During the next 48 h of hyperoxia exposure, 15% and 20% of the mice in the MEFCs- and BM-MSCs- treated groups died, respectively. After 84 h post-hyperoxia exposure, 30%, 33% and 63% of the mice died in the PBS placebo, MEFCs-treated, and BM-MSCs-treated groups, respectively, but 100% survival rate in the AFSCs-treated group. After 102 h post-hyperoxia exposure, the mice that had received intravenous AFSCs therapy had a significant higher survival rate (90%) than the mice that had received intravenous MEFCs (60%), BM-MSCs (37%), and PBS (30%) (p<0.01; Figure 3). The survival rate in the AFSCs-treated mice was stably maintained at 43% from 114 h to 14 days post-hyperoxia exposure, but only 27%, 10%, and 0% survival rates were obtained in the BM-MSCs-, PBS-, and MEFCs-treated groups, respectively, during the longer-term observation.

**Figure 4:**
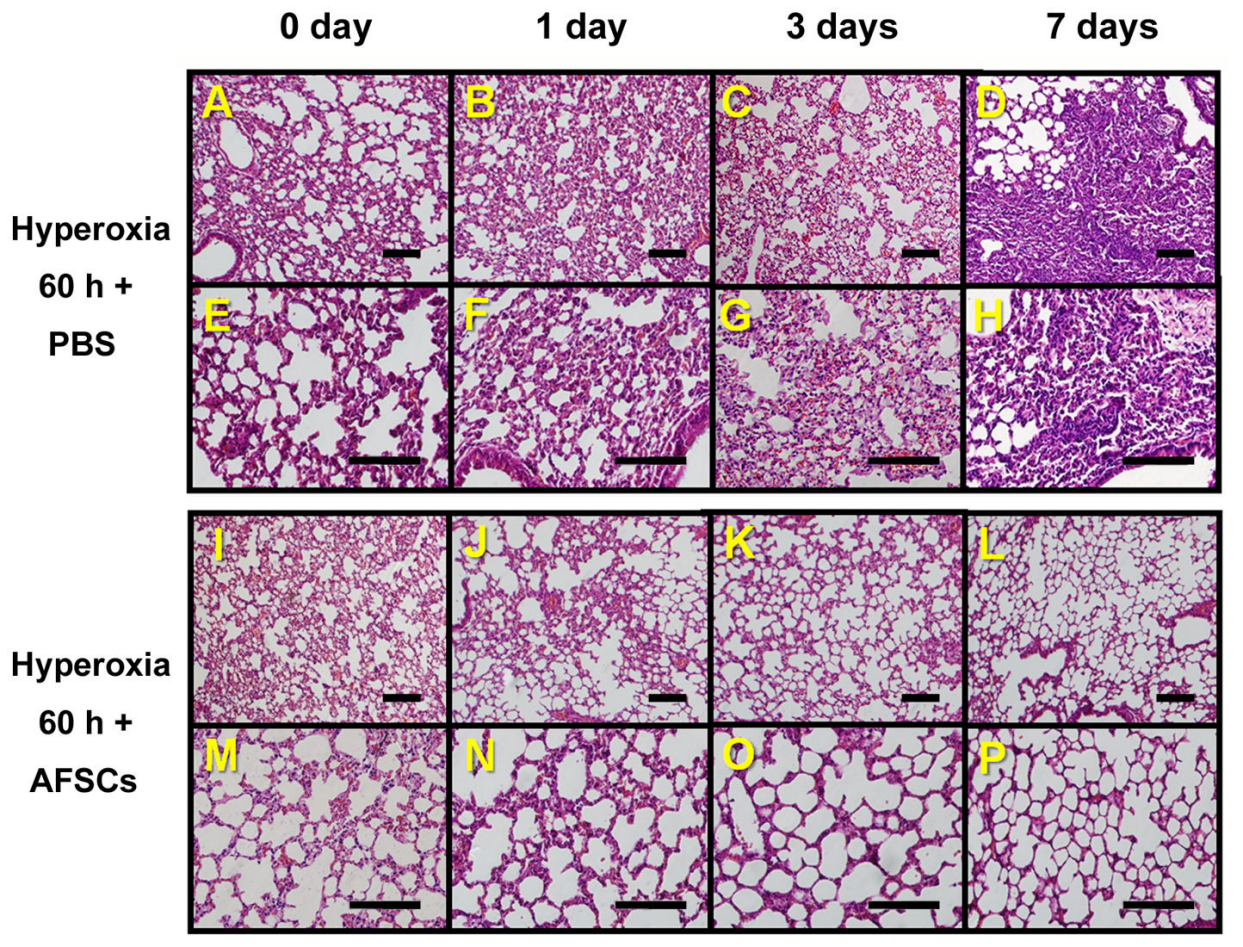
Histological comparisons of lung tissues from the hyperoxia-exposed control mice and the hyperoxia-exposed AFSCs-treated mice. (**A**, **B**, **C**, **D**) Lung histology (H&E staining, 200x magnification) of mice exposed to hyperoxia for 60 h, shown at 0, 1, 3, and 7 days, respectively, after treatment with PBS. (**E**, **F**, **G**, **H**) The same groups as above shown at 400x magnification. (**I**, **J**, **K**, **L**) Histology (H&E staining, 200x magnification) of lung sections from animals exposed to hyperoxia for 60 h, shown at 0, 1, 3, and 7 days, respectively, after treatment with AFSCs. (**M**, **N**, **O**, **P**) The same groups as shown in **I, J, K**, and **L** shown at 400x magnification. Scale bar =100 µm.

Lung tissue was harvested on days 1, 3, and 7 after 60-hour hyperoxia. The obvious pathological differences between the control and treated groups were began at the day 1 post-hyperoxia, at which point the H&E staining of lung sections from AFSCs-treated mice had significant less lung injury (inflammatory cell infiltration and alveolar wall destruction, Figure 4I–P) than the control mice given PBS (Figure 4A–H).

### Effect of AFSCs on lung edema, inflammation, apoptosis, and fibrosis induced by hyperoxia

**Figure 5:**
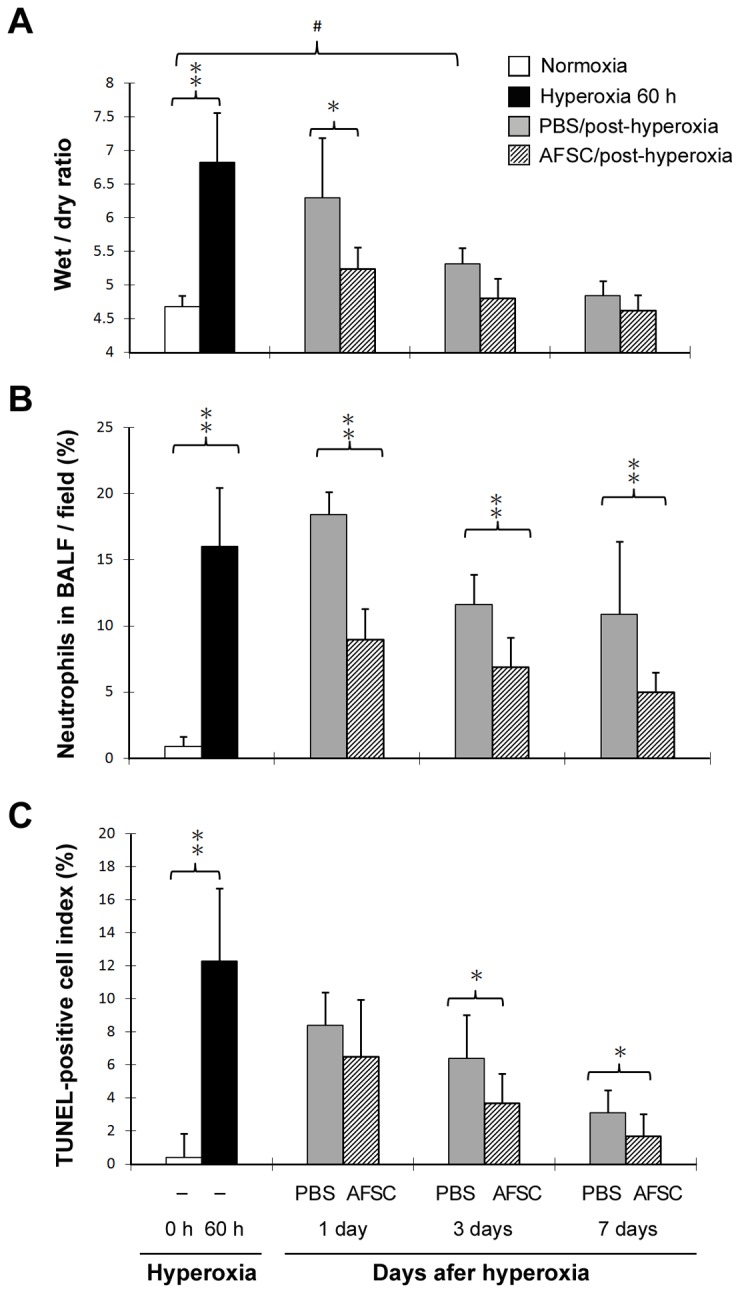
Comparison of the wet/dry ratio, neutrophil infiltration, and TUNEL assay in lungs from the hyperoxia-exposed PBS-treated control mice and the AFSCs-treated mice. (**A**) Pulmonary edema was measured as the wet/dry weight ratio of the lungs (n=10 in each group). (**B**) Pulmonary inflammation measured as the leukocyte counts of the BALF (n=10 in each group). (**C**) Pulmonary cell apoptosis measured as the TUNEL assay of the lungs (n=10 in each group). **p<0.01; *p<0.05; ^#^ p<0.05.

As expected, the mice exposed to 60 h hyperoxia had a significantly higher wet/dry ratio than the control mice (6.7 vs. 4.7, p<0.01). Furthermore, one day after hyperoxia exposure, the mice that received the AFSCs treatment had a significantly lower wet/dry ratio (5.2 vs. 6.3, p<0.05) than the mice without AFSCs treatment (Figure 5A). To determine the effect of AFSCs on the inflammatory response, BALF was analyzed for neutrophil content. The mice exposed to hyperoxia had significantly higher levels of neutrophil infiltration as measured in the cytospin slide than the control mice (16% vs. 2%, p<0.01). Neutrophil infiltration was significantly less in the mice that received the AFSCs treatment than in the mice that received PBS (p<0.01), at all three post-hyperoxia periods (days 1, 3, or 7 post-hyperoxia) (Figure 5B).

**Figure 6:**
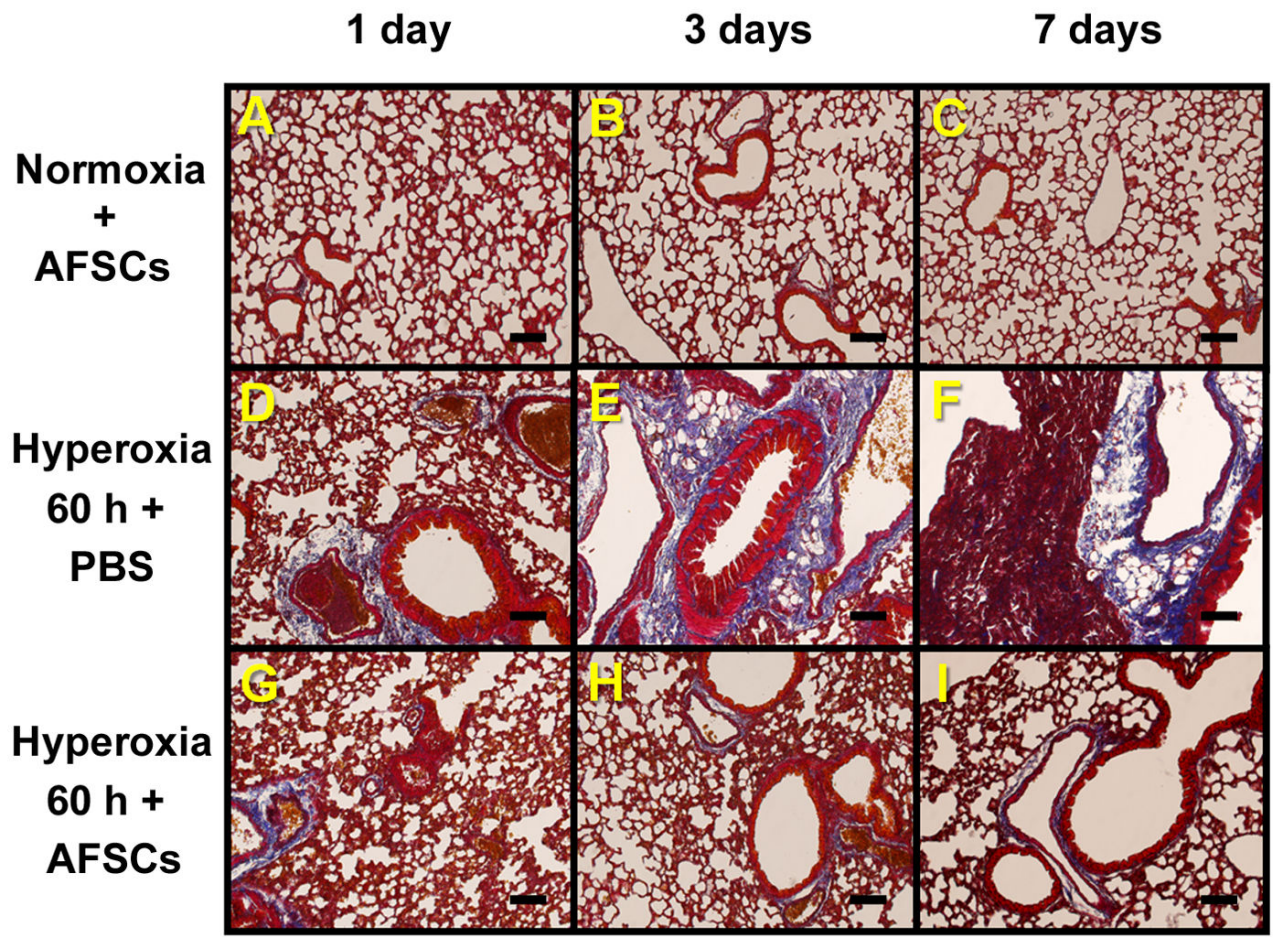
Comparison of fibrosis status using Masson trichrome stain in lungs from the hyperoxia-exposed PBS- or AFSCs-treated mice. (**A**, **B**, **C**) The normoxia-exposed mice treated with AFSCs at 1, 3, and 7 days, respectively. (**D**, **E**, **F**) The hyperoxia-exposed mice treated with PBS at 1, 3, and 7 days, respectively. (**G**, **H**, **I**) The hyperoxia-exposed mice treated with AFSCs at 1, 3, and 7 days, respectively. Scale bar =100 µm.

The TUNEL assay was used to determine whether AFSCs influence the apoptosis of the lung cells in the hyperoxia model. The mice exposed to hyperoxia had significantly higher percentages of TUNEL-positive cells than the mice exposed to room air (12% vs. 0.5%, p<0.01). The mice that received AFSCs had significantly fewer TUNEL-positive cells than the mice that received PBS days 3 and 7 after the 60 h period of hyperoxia (Figure 5C). Masson trichrome staining indicated that the normal lung images were shown in Figure 6A–C and the mice that received PBS at days 3 and 7 post-hyperoxia exposure (Figure 6D–F) had significantly severer fibrosis than the mice that received the AFSCs treatment (Figure 6G–I).

### AFSCs reduced inflammatory cytokines in mice lung tissues

**Figure 7:**
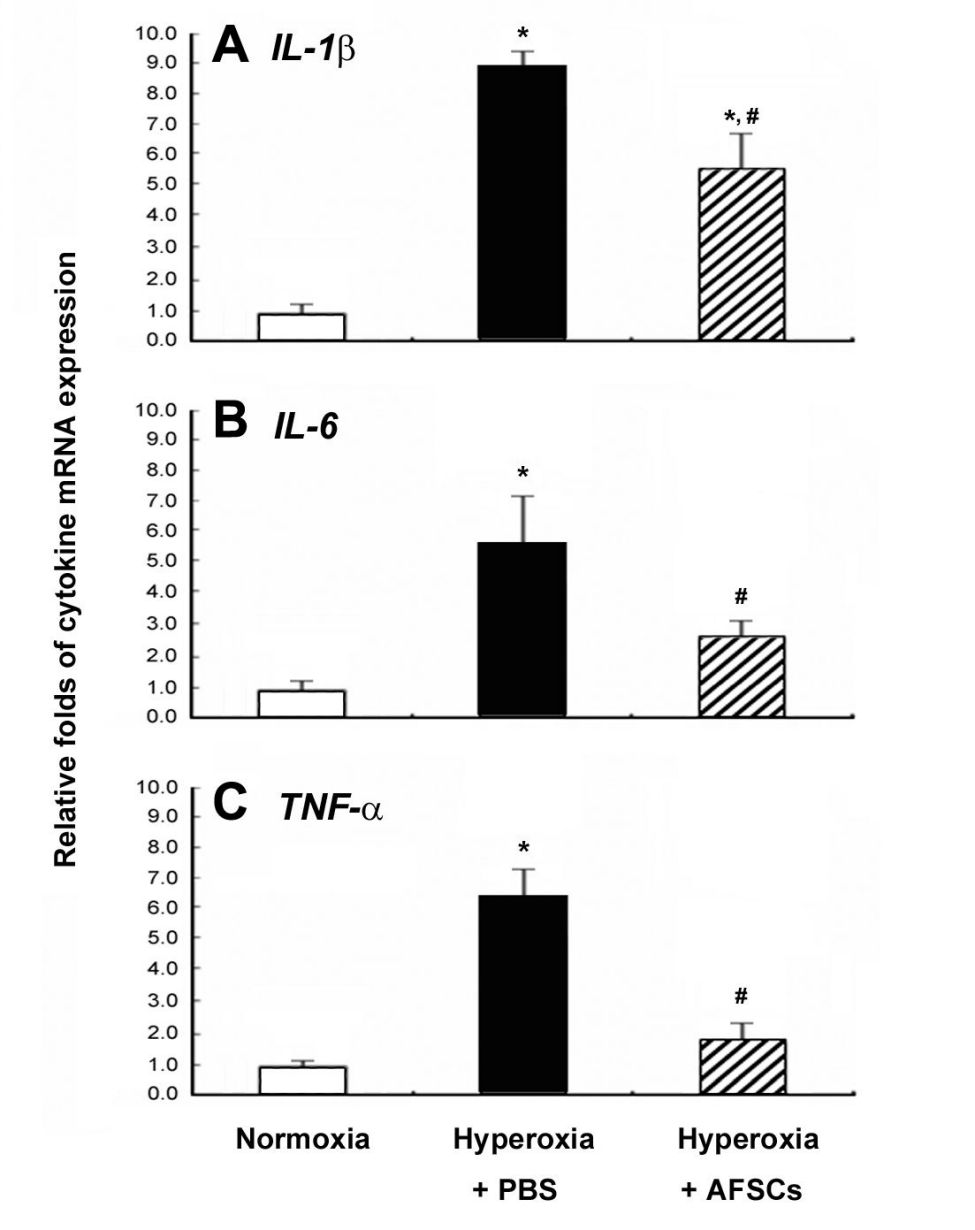
Comparison of inflammatory cytokine mRNA expression levels in the lungs of the hyperoxia-exposed PBS-treated control mice and the AFSCs-treated mice. IL-1β (**A**), IL-6 (**B**), and TNF-α (**C**) mRNA expression levels were measured using quantitative RT-PCR from the following three groups: normoxia mice, hyperoxia-exposed mice treated by PBS, and hyperoxia-exposed mice treated by AFSCs at day 7. *p<0.05 vs. normoxia group; ^#^ p<0.05 vs. hyperoxia-exposed with PBS group.

To further evaluate the anti-inflammatory actions by AFSCs, quantitative real-time RT-PCR was used to measure the levels of proinflammatory cytokines expressions in the lung tissues of the mice (Figure 7). Inflammatory cytokines (IL-1β, IL-6, and TNF-α) were all elevated in lung tissues in response to hyperoxia exposure (8.9 ± 0.6, 5.6 ± 1.7, and 6.4 ± 0.9 folds, respectively) compared with normoxia (p<0.05). The treatment with AFSCs significantly decreased the levels of proinflammatory cytokine expression (5.5 ± 1.2, 2.7 ± 0.4, and 1.9 ± 0.5 folds, respectively) compared with PBS treatment (p<0.05).

### Accumulation of AFSCs within the inflammatory site of the injured lung

**Figure 8:**
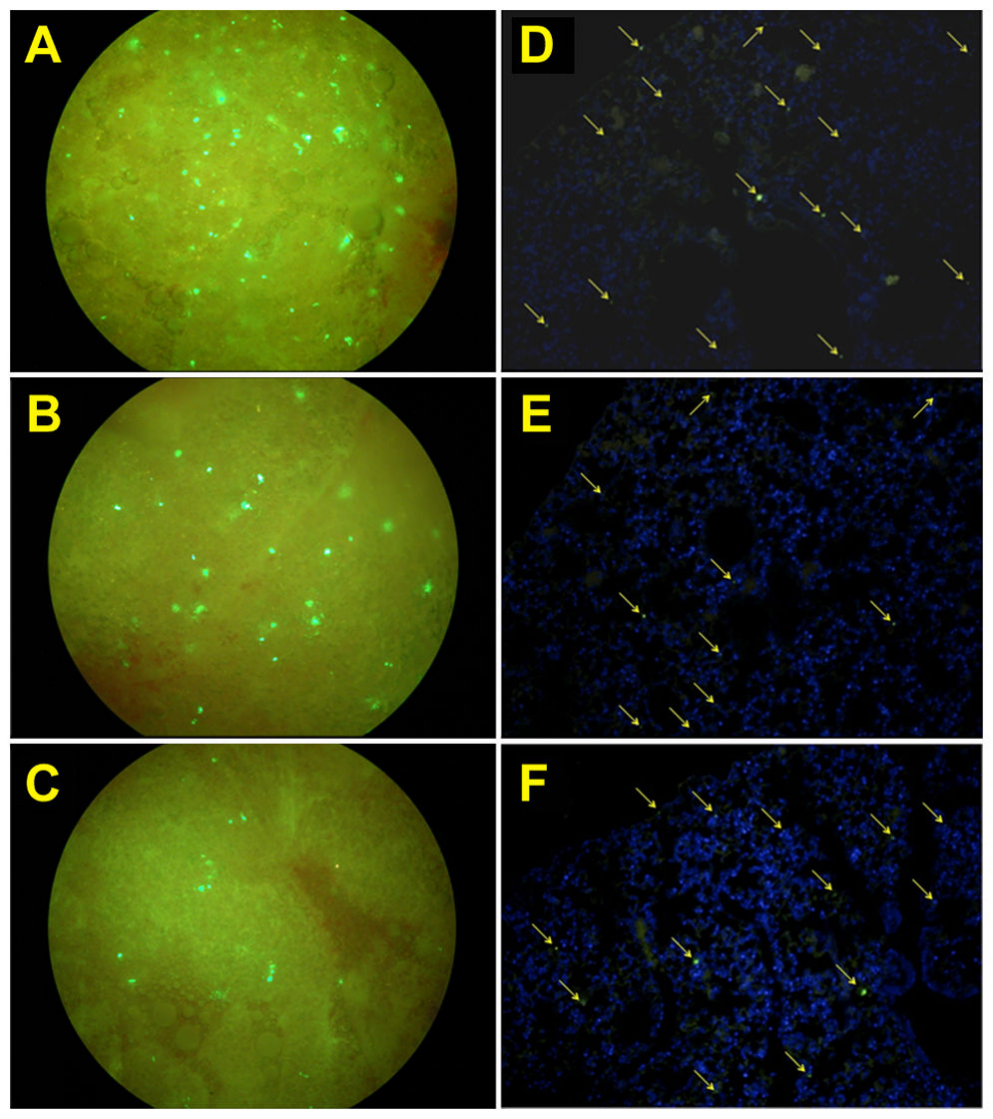
Migration of the intravenously injected EGFP-expressing AFSCs to lung tissue after hyperoxia exposure for 60 h. (**A**, **B**, **C**) The lung tissues were minced and examined by fluorescence microscopy at 200x magnification 1, 3, and 7 days, respectively, after AFSCs were injected. (**D**, **E**, **F**) The paraffin-embedded lung sections were stained with DAPI and immunostained for EGFP-positive AFSCs 1, 3, and 7 days, respectively, after injection of the AFSCs, shown at 200x magnification. Scale bar = 100 µm.

To determine whether EGFP-expressing AFSCs could accumulate within the lung tissue, we analyzed the EGFP-expressing cells in the lung sections obtained on days 1, 3, and 7 after hyperoxia-exposure. Fluorescence microscopy revealed that the numbers of EGFP-expressing cells were greatest on day 1 after hyperoxia-exposure (Figure 8A). As shown in Figure 8, the numbers of EGFP-expressing cells gradually decreased on the following days (Figure 8A, 8B, and 8C) relative to the DAPI-stained cell populations (Figure 8D, 8E, and 8F).

### Conditioned medium from hyperoxia-exposed lung tissues increased AFSCs migration

**Figure 9:**
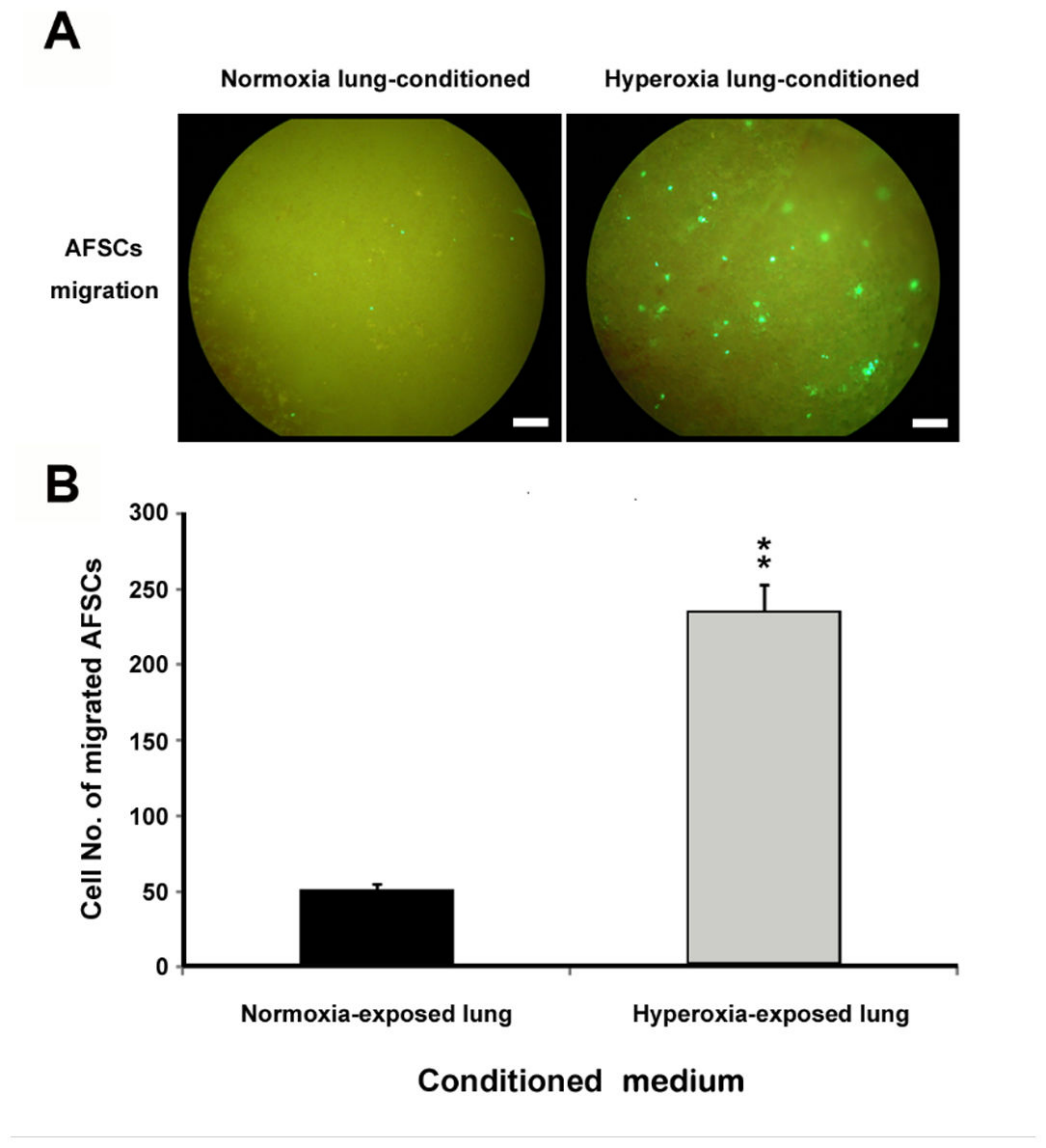
Migration of AFSCs is induced by hyperoxia-exposed lung tissues. (**A**) AFSCs migration assay using the transwell system. A total of 5x10^3^ AFSCs were seeded in the inner chamber and minced lung tissues from mice exposed to normoxia (left) or 60 h of hyperoxia (right) were placed in outer chambers. (**B**) The EGFP-expressing AFSCs that had migrated to the outer chambers were quantified. **p<0.01. Scale bar = 50 µm.

To confirm whether AFSCs has the ability to migrate to the inflammatory sites *in vitro*, we performed co-culture of hyperoxia-exposed lung tissues and AFSCs (Figure 9A). In this experiment, the medium from hyperoxia-exposed lung tissues significantly increased the numbers of migrating AFSCs more than 5-fold relative to the numbers that migrated in response to the control lung-conditioned medium (p<0.01; Figure 9B).

## Discussion

The results of this study are the first to demonstrate that AFSCs from EGFP transgenic mice attenuates lung injury in mice with hyperoxia-induced ALI. Engraftment of the AFSCs reduced the lung water content, neutrophil infiltration, apoptosis and pulmonary fibrosis, and levels of inflammatory cytokines, suggesting that the therapeutic benefits of AFSCs in ALI are likely to be the result of their anti-inflammatory and immune-modulating effects.

Oxygen supplementation is frequently used in critically ill patients, especially those with ALI [36] or cardiopulmonary diseases. Short-term exposure to hyperoxia (48-72 h) has been shown to induce ALI, whereas prolonged exposure (>96–120 h) causes death in rodents [8]. Thus, in the present study, ALI produced by a relatively short (60 h) period of hyperoxia exposure was used as a model to investigate the mechanisms that control lung injury, repair, and inflammation. Previous studies have shown that exposure to hyperoxia for 60–72 h causes damage to lung alveolar epithelial and endothelial cells, resulting in increased vascular and alveolar permeability and inflammatory cellular infiltration [10,37]. Therefore, it is crucial to focus the therapeutic goals toward regeneration of these cell types after lung injury [38].

Regarding regeneration of the injured lung cells, exogenous stem cells have shown potential as cellular therapeutic agents to enhance lung repair mechanisms. However, embryonic stem cell research has been hampered by moral objections to the destruction of embryos required to harvest the cells. In contrast, amniotic fluid stem cells appear to have many of the key therapeutic benefits of embryonic stem cells while avoiding their significant ethical, medical, and logistical drawbacks. In the present study, we isolated AFSCs with basic mesenchymal stem cell (MSC) characteristics from EGFP transgenic mice. The isolated cell population exhibited typical characteristics: fibroblastic morphology, clonogenic capacity, multipotential differentiation capability and expression of a typical set of surface antigens [39–41]. Although no single specific marker is known for murine MSC, previous studies have demonstrated the strong expression of CD44 by murine AFSC [42–44]. To determine the ability of AFSC to migrate to mice lung, we injected EGFP-expressing AFSCs from EGFP transgenic mice into non-transgenic animals and used fluorescence microscopy to identify the injected cells in the lung at different time points. We confirmed that AFSCs were present in the hyperoxia-injured lungs 24 h after injection. During the following days, the AFSCs circulated throughout the body (data not shown) and EGFP-positive cells in the lung gradually diminished (Figure 8). To further evaluate whether lung damage plays a crucial role in attracting AFSCs to injured lungs, we performed a co-culture experiment to expose the AFSCs to conditioned medium from control or hyperoxia-exposed lung tissue. We found that medium from damaged lungs promoted the migration of significantly greater numbers of AFSCs than that of normal lung. This result is consistent with previous findings [45], that showed that accumulation of inflammatory mediators could attract stem cells [26].

Notably, intravenously injected AFSCs reduced the edema, neutrophil infiltration, apoptosis in the lungs and early fibrosis at 7 days post-hyperoxia (Figure 5). These results suggest that the early modulation of inflammation by AFSCs leads to an attenuation of the downstream events that cause collagen deposition and fibrosis. The reduction of inflammatory cytokine expression at 7 days after AFSCs injection further indicates the immunomodulatory effects of AFSCs in the hyperoxia-injured lung. The immunomodulatory effects of BM-MSCs have been studied in a variety of inflammatory conditions including graft-versus-host disease and autoimmune diseases [46,47]. In this study, we found that AFSCs is the best repair cells to improve mice survival rate after hyperoxia-induced ALI compared with BM-MSCs- and MEFCs-treated groups (Figure 3). For the safety test of AFSCs, mice monitored up to 3 months from the intravenous injection did not show any neoplasia arising from the EGFP-expressing AFSCs. Also, intramuscular injection of AFSCs in the recipient mice did not produce any tumor formation during 3 months observation (data not shown). The same results have been shown previously that fetal mesenchymal stromal cells can be extensively expanded from amniotic fluid, showing no karyotypic abnormalities or transformation potential *in vitro* and no tumorigenic effect *in vivo* [48,49]. The utilization of AFSCs for tissue repair and regeneration offers advantages over the use of ESCs or adult BM-MSCs: (1) AF represents a convenient and non-argued source for obtaining stem cells; (2) their derivation is relatively simple and rapid; (3) no feeder layers are required for their cultivation; and (4) their stem cell phenotype is not affected by long-term storage [24]. Therefore, the application of AFSCs for tissue repair or replacement therapies is a great potential for clinical use.

Several studies have shown that AFSCs are an effective viable source in regenerative medicine. In experimental studies, AFSCs have be successfully used in the treatment of a variety of diseases, such as large-scale skin wounds and burns [50], injured urethral sphincter [51], chronic allograft vasculopathy [52], and neural tube defects [53]. A recent study also showed that the intravenous grafts of AFSCs induced endogenous cell proliferation in ischemic stroke rats [54]. In clinical application, AFSCs used in the treatment of acute stroke by intravenous route of transplantation has been developing.

In conclusion, this study demonstrates that intravenous injection of AFSCs in an experimental model of hyperoxia-induced ALI provides a significant survival advantage that is associated with a reduction of lung edema, neutrophil infiltration, apoptosis, cytokine expression, and lung fibrosis. AFSCs may offer a new therapeutic strategy for acute lung injury (ALI), for which efficient treatments are currently unavailable.
